# Differential therapeutic effects of PARP and ATR inhibition combined with radiotherapy in the treatment of subcutaneous versus orthotopic lung tumour models

**DOI:** 10.1038/s41416-020-0931-6

**Published:** 2020-06-17

**Authors:** Vanessa Tran Chau, Winchygn Liu, Marine Gerbé de Thoré, Lydia Meziani, Michele Mondini, Mark J. O’Connor, Eric Deutsch, Céline Clémenson

**Affiliations:** 1 INSERM U1030, Molecular Radiotherapy, Gustave Roussy Cancer Campus, Université Paris-Saclay, Villejuif, France; 2 Labex LERMIT, DHU TORINO, SIRIC SOCRATE, Villejuif, France; 3 Oncology Innovative Medicines and Early Clinical Development, AstraZeneca, Cambridge UK; 4 Department of Radiation Oncology, Gustave Roussy Cancer Campus, Villejuif, France

**Keywords:** Cancer models, Lung cancer, Drug safety

## Abstract

**Background:**

Subcutaneous mouse tumour models are widely used for the screening of novel antitumour treatments, although these models are poor surrogate models of human cancers.

**Methods:**

We compared the antitumour efficacy of the combination of ionising radiation (IR) with two DNA damage response inhibitors, the PARP inhibitor olaparib and the ATR inhibitor AZD6738 (ceralasertib), in subcutaneous versus orthotopic cancer models.

**Results:**

Olaparib delayed the growth of irradiated Lewis lung carcinoma (LL2) subcutaneous tumours, in agreement with previous reports in human cell lines. However, the olaparib plus IR combination showed a very narrow therapeutic window against LL2 lung orthotopic tumours, with nearly no additional antitumour effect compared with that of IR alone, and tolerability issues emerged at high doses. The addition of AZD6738 greatly enhanced the efficacy of the olaparib plus IR combination treatment against subcutaneous but not orthotopic LL2 tumours. Moreover, olaparib plus AZD6738 administration concomitant with IR even worsened the response to radiation of head and neck orthotopic tumours and induced mucositis.

**Conclusions:**

These major differences in the responses to treatments between subcutaneous and orthotopic models highlight the importance of using more pathologically relevant models, such as syngeneic orthotopic models, to determine the most appropriate therapeutic approaches for translation to the clinic.

## Background

Radiotherapy (RT) is a mainstay of current anticancer strategies. One of its limitations lies in its toxicity to normal tissues that are close to the radiation field.^1^ In addition, accumulating preclinical and clinical evidence indicates that the microenvironment and the immune system modulate the antitumour efficacy of cytotoxic treatments, such as RT.^2–4^ Thus, the proper preclinical evaluation of novel therapeutic strategies that include RT should be performed with models recapitulating at best the ‘physiological' tumour environment, comprising the pertinent tumour stroma, an intact immune system and the presence of the appropriate surrounding healthy tissues. For years, subcutaneous tumour models implanted in immunocompromised mice have been a standard for the preclinical evaluation of novel drugs and therapeutic combinations without filling this requirement, and possibly leading to incorrect assessments for translation into the clinic. Preclinical data in tumours grafted orthotopically in immunocompetent hosts are sparse.

Radiotherapy (RT) exerts its cytotoxic effect by inducing DNA damage. Cells have evolved to respond to extensive DNA damage through sophisticated cell-cycle checkpoints and DNA repair pathways. Inhibiting the DNA damage response (DDR) in tumour cells is a rational strategy to augment the cytotoxicity of RT.^5^ Poly(ADP-ribose) polymerase 1 (PARP-1) is a nuclear protein involved in base-excision repair (BER), and is critical for the repair of single-strand breaks (SSBs) and single-strand intermediates.^6,7^ During genome duplication, the collision of replication forks with unrepaired SSBs and/or trapped PARP–DNA complexes, resulting from PARP inhibition, leads to the formation of potentially lethal DNA double-strand breaks (DSBs), the repair of which is highly dependent on homologous recombination (HR).^6^ Thus, HR-deficient cells are extremely sensitive to PARP inhibition.^8^ This synthetic lethality has been extensively investigated in BRCA-mutated ovarian cancer, leading to the approval of the PARP inhibitor olaparib in over 60 countries. However, the efficacy of PARP inhibitors (PARPi) in the clinic may be limited by the emergence of resistance. The restoration of HR competency and replication fork stabilisation (fork protection) has been described as two critical compensatory PARPi- resistance mechanisms.^9,10^ As the damage induced by PARP inhibition is generated during the S phase, PARPi-treated cells rely heavily on the DDR protein ATR (ataxia telangiectasia and Rad3-related), which plays a major role in survival during DNA replication stress.^9,10^ ATR inhibition significantly enhanced the cytotoxicity of PARPi, not only in BRCA-mutated but also in BRCA-proficient and PARPi-resistant human cancer cells.^10–13^

Numerous preclinical studies have previously demonstrated that PARP or ATR inhibition radiosensitises human cancer cells in vitro, and improves RT efficacy in vivo in human tumour models xenografted subcutaneously into immunodeficient mice.^14–31^ In this study, LL2-luc Lewis lung carcinoma murine tumour cells were implanted either subcutaneously or orthotopically. Established tumours were treated with combination treatments of ionising radiation (IR), PARP inhibitor (olaparib) and ATR inhibitor (AZD6738/ceralasertib). As expected, olaparib or AZD6738 radiosensitised LL2-luc cells in vitro, and improved the efficacy of radiotherapy against LL2-luc subcutaneous tumours in vivo. The triple-combination treatment (IR + olaparib + ceralasertib) delayed tumour growth even further. However, the olaparib plus IR combination treatment showed limited efficacy against LL2-luc orthotopic tumours, and signs of toxicity were revealed. The addition of AZD6738 to this therapeutic combination did not augment the antitumour efficacy. In addition, mucositis was observed in a head and neck orthotopic model treated with this triple combination. Thus, our study highlights different responses to antitumour treatments and different therapeutic windows between subcutaneous and orthotopic tumour model settings, warranting the need to define and systematically use clinically relevant preclinical tumour models.

## Methods

### Cells and reagents

LL2-luc cells were purchased from Caliper Life Sciences (Hopkinton, MA, USA). TC1-luc cells generated by the HPV16 E6/E7 and c-H-ras retroviral transduction of lung epithelial cells of C57BL/6 origin were kindly provided by T.C. Wu (Johns Hopkins Medicine, Baltimore, MD, USA). The following antibodies were used: anti-Poly-ADP Ribose (Trevigen 4336-BPC-100, D1/1000), anti-PARP-1 (CST#9532, D1/1000), anti-GAPDH (MAB374, D1/10000), anti-p-Chk1 (CST#2348, D1/1000), anti-Chk1 (CST#2360, D1/1000), anti-actin (MAB1501, D1/100000) and secondary antibodies (SouthernBiotech 4050-05 and 1031-05, D1/5000). For protein extraction, cells were harvested in SDS–urea buffer for PAR staining, or otherwise harvested in RIPA buffer containing protease and phosphatase inhibitors (Complete and PhoSTOP, Roche (Basel, Switzerland)). Olaparib and AZD6738 were provided by AstraZeneca. H_2_O_2_ was purchased from Sigma-Aldrich. Cells were irradiated with an X‐ray XRAD320 tube (320 kV, 12.5 mA).

### Proliferation and clonogenic assays

For proliferation assays, cells were treated with various doses of drug(s), and a WST-1 assay was conducted 72 h later, according to the manufacturer’s instructions (Roche, France). Cells were incubated with the WST-1 reagent, and the absorbance, which is correlated to the number of viable cells, was measured with a spectrophotometer. For clonogenic assays, cells were plated at a single-cell density, treated with the drug(s), and then irradiated or not 1 h later. The colonies were stained with crystal violet. The surviving fraction (SF) was calculated as follows: SF = number of colonies formed/number of cells seeded × plating efficiency of the nonirradiated group.

### In vivo mouse experiments

Animal procedures were performed according to protocols approved by the Ethics Committee CEEA26 (project no. 2015‐016‐613, EU directive 2010/63/EU). Female C57BL/6 mice (7–8-weeks old, ~20 g) were purchased from Janvier (France) and housed in the Gustave Roussy animal facility (SPF, animal care license no. D94-076-11). Mice were used after an acclimation time of 7 days. They had free access to food (SAFE reference R0340, Augy, France) and water. They were housed on a 12-h light/dark cycle at a room temperature of 22 °C ± 2 °C and a relative humidity of 55% ± 15% (five animals per cage, disposable ventilated cages, Innovive, France). Irradiation was performed locally to the subcutaneous tumour, the whole thorax or the snout with an X‐ray XRAD320 tube (320 kV, 12.5 mA, dose rate of 1.08 Gy/min), the rest of the body being protected by a lead shield. Olaparib and AZD6738 were administered intraperitoneally 1 h before irradiation in the morning. Olaparib and AZD6738 were formulated in 10% DMSO + 10% kleptose in purified sterile water, and in 10% DMSO + 40% propylene glycol + 50% sterile water, respectively. The control groups were injected with these vehicles and were sham-irradiated. We chose to administer olaparib and AZD6738 at doses previously used in combination with radiation in preclinical studies.^20,23,25,28,31^

For subcutaneous grafts, 600,000 LL2-luc cells (in PBS, 50 µl) were injected into the right flank. When the tumours reached ~80 mm^3^, the mice were allocated to different treatment groups (day 1). The tumour size was measured with an electronic calliper. The tumour volume was estimated from two-dimensional tumour measurements (volume = length × width^2^/2). The endpoint for survival was a tumour exceeding 500 mm^3^.

For orthotopic lung tumours, under anaesthesia (2% isoflurane), the skin was incised, 600,000 LL2-luc cells (in PBS + Matrigel (BD#356231), 10 µl) were injected directly into the lung through the pleura and then the wound was closed by suture clips. Tumour growth was monitored by bioluminescence imaging using the Xenogen In Vivo Imaging System 50 (Caliper Life Sciences) under anaesthesia (2% isoflurane).^32^ For head and neck tumours, TC1-luc cells were injected at a submucosal site in the right inner lip (500,000 cells in 50 µl) under anaesthesia (2% isoflurane).^33^ Tumour growth was monitored using bioluminescence imaging. For both orthotopic models, the ethical endpoint for survival was loss of more than 20% of the initial weight or severe clinical symptoms.

All animals were killed by cervical dislocation. All the experimental procedures were conducted in a laboratory.

### Statistical analysis

Clonogenic survival is depicted as the mean ± SEM. Statistical analyses were performed using GraphPad Prism software (ns, *P* > 0.05; *P < 0.05; **P < 0.01; ***P < 0.001 and ****P < 0.0001). Survival data were analysed using the Kaplan–Meier method, and compared statistically using the log-rank test (the Bonferroni correction was used to adjust for multiple comparisons). Tumour growth (calliper measurement or bioluminescence) was analysed using a two-way ANOVA followed by Tukey’s post hoc test for multiple comparisons (tables present all the comparisons that have been evaluated).

## Results

### Olaparib enhances the effect of IR against subcutaneous murine LL2-luc syngeneic tumours

**Fig. 1:**
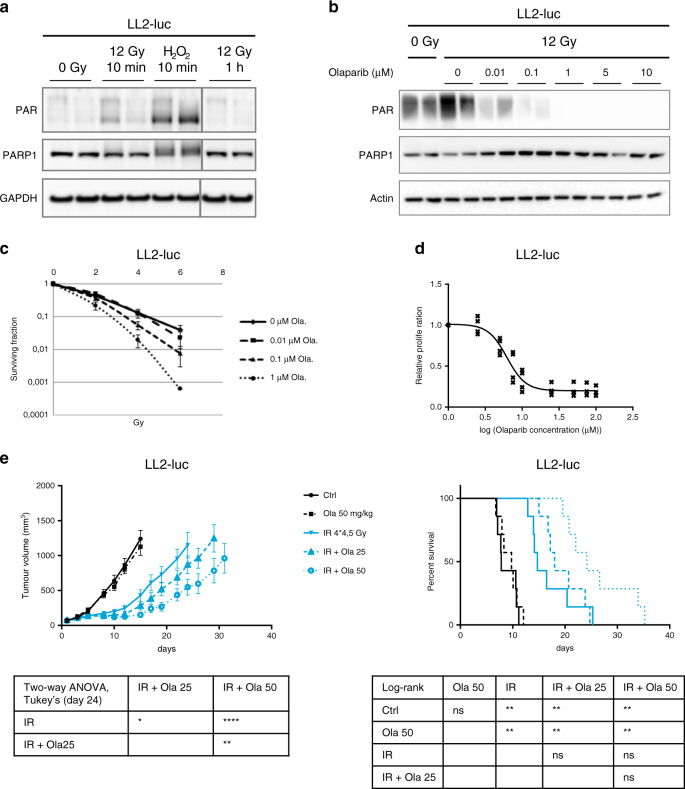
The PARP inhibitor olaparib increases the efficacy of ionising radiation against subcutaneous murine syngeneic Lewis lung LL2-luc tumours. **a** Western blot analysis of PAR formation 10 min and 1 h after a 12-Gy dose irradiation in LL2-luc cells. Extensive PARylation was observed in cells treated for 10 min with the positive control H_2_O_2_ (1 mmol/L). **b** Olaparib treatment inhibits radio-induced PAR formation. LL2-luc cells were treated with olaparib for 1 h before irradiation (12 Gy). Protein extracts were prepared at 10 min after irradiation. **c** Clonogenic survival of LL2-luc cells exposed to olaparib (for 24 h) and irradiated 1 h after initiating olaparib treatment. **d** LL2-luc cells were treated with olaparib for 72 h, and proliferation was analysed by WST-1 assay. **e** Tumour growth (top-left panel) of LL2-luc subcutaneous syngeneic grafts (mean ± SEM). Mice-bearing LL2-luc subcutaneous tumours were allocated to treatment groups on day 1 (*n* = 7 mice per group). Olaparib (25 mg/kg or 50 mg/kg) was administered daily from day 1 to day 6. Irradiated mice received four fractions of 4.5 Gy (on days 1, 2, 4 and 5). Kaplan–Meier analysis of mouse survival (top right) and statistical analyses of tumour growth and survival (bottom left and bottom right, respectively) are depicted.

As observed in human cell lines,^14,34^ IR induced a moderate and transient increase in PAR levels in vitro in the BRCA wild-type, murine LL2-luc luciferase-expressing Lewis lung carcinoma cell line (Fig. 1a). PARP inhibition eradicated radio-induced PAR formation at concentrations above 0.1 µmol/L (Fig. 1b), warranting the investigation of the combination of PARP inhibition with IR. A radiosensitising effect of olaparib in the clonogenic assay was observed at these concentrations (SER_0.37_ = 1.68 for 1 µmol/L olaparib) (Fig. 1c), while olaparib inhibited LL2-luc cell proliferation at higher concentrations (IC_50_: 6.2 µmol/L) (Fig. 1d). Olaparib alone did not delay tumour growth in vivo, in accordance with previous studies,^20,21,28^ but greatly improved the efficacy of IR (4 fractions of 4.5 Gy) against LL2-luc subcutaneous tumours implanted in syngeneic immunocompetent mice (Fig. 1e).

### The efficacy of the olaparib plus IR combination treatment against the LL2-luc lung orthotopic tumour model is limited

**Fig. 2:**
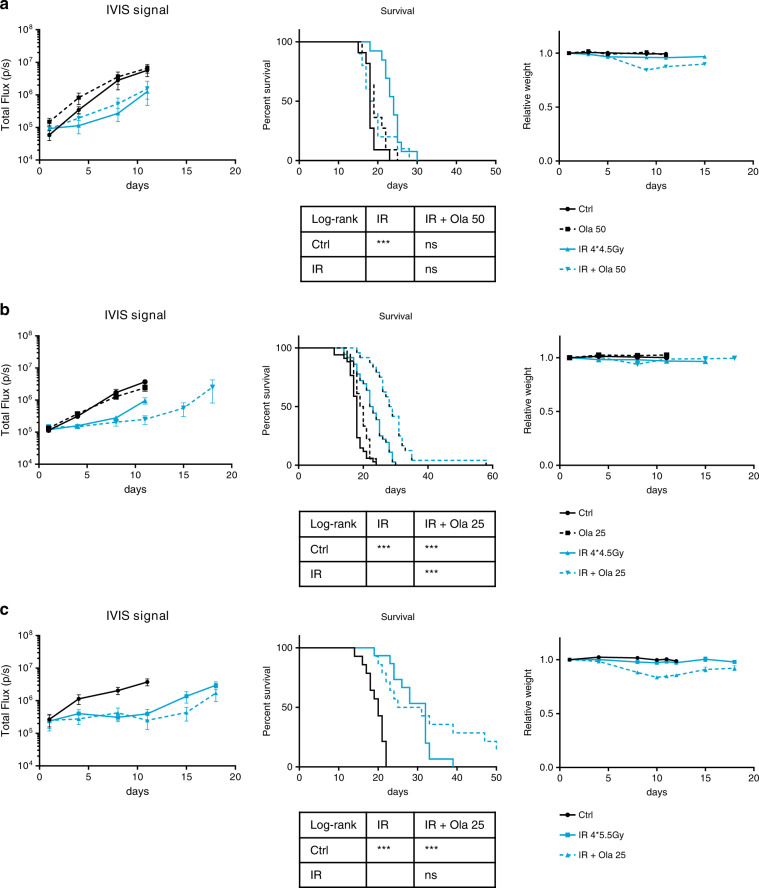
Limited efficacy of the olaparib plus irradiation combination treatment against lung LL2-luc orthotopic tumours. Three days after the orthotopic implantation of LL2-luc cells into the lung, the mice were allocated to treatment groups (day 1). Olaparib was administered daily from day 1 to day 6. Irradiated mice received four fractions on days 1, 2, 4 and 5. Three different treatment conditions were evaluated: a combination of 50 mg/kg olaparib with 4 fractions of 4.5 Gy (**a**), a combination of 25 mg/kg olaparib with 4 fractions of 4.5 Gy (**b**) and a combination of 25 mg/kg olaparib with four fractions of 5.5 Gy (**c**). For each condition, tumour growth monitored by bioluminescence imaging (mean ± SEM, left panel), Kaplan–Meier analysis of mouse survival (middle panel), relative mouse weight (mean ± SEM, right panel) and statistical analysis of survival (bottom) are depicted (**a**, *n* = 10–13; **b**, *n* = 18–36; **c**, *n* = 12–15).

To implant orthotopic tumours, LL2-luc cells were directly injected into the lung as previously described,^32^ and tumour growth was followed by bioluminescence imaging. Surprisingly, the antitumour efficacy of IR was not enhanced by concomitant treatment with 50 mg/kg olaparib (Fig. 2a). The survival of the combination-treated mice tended to be shorter than that of the irradiated mice (Fig. 2a, middle panel). Notably, the mice treated with the combination treatment showed signs of prostration, weakness and weight loss (Fig. 2a, right panel), revealing toxicity issues, and the mice that experienced severe clinical signs had to be sacrificed. When the olaparib dose was reduced to 25 mg/kg, no weight loss was observed in the combination treatment group (Fig. 2b, right panel), and compared with irradiation alone, the combination treatment improved mouse survival and inhibited tumour growth (Fig. 2b). However, this enhancement of IR antitumour activity was abolished when mice were irradiated with a higher dose of irradiation (4 fractions of 5.5 Gy, Fig. 2c), and a body weight loss similar to that detected in the IR 4*4.5 Gy + olaparib 50 mg/kg treatment group was observed (Fig. 2a, c, right panels). The lack of efficacy was not due to impaired PARP inhibition in orthotopic tumours since olaparib treatment at doses as low as 25 mg/kg did inhibit PAR formation in tumours (Supplementary Fig. 1). Orthotopic tumours are smaller than subcutaneous tumours at treatment initiation (Supplementary Fig. 2). As small tumours are generally more sensitive to antitumour treatment, it seems rather unlikely that the different response of orthotopic versus subcutaneous tumours is due to the difference in tumour size at treatment initiation. Our results suggest that the efficacy of the olaparib plus IR combination in the orthotopic tumour model is limited by the toxicity induced by high doses of irradiation or olaparib.

**Fig. 3:**
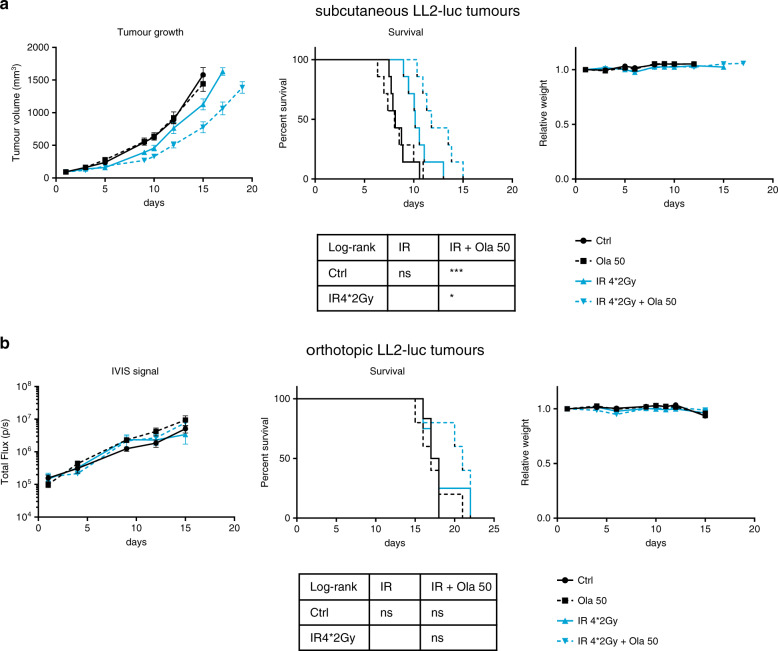
At a lower dose of irradiation (4*2 Gy), the olaparib and irradiation combination treatment is effective against subcutaneous tumours but not against orthotopic tumours. Subcutaneous (*n* = 7) (**a**) or orthotopic (*n* = 5–6) (**b**) LL2-luc tumours were allowed to grow. Then, the mice were randomised (day 1) and treated with olaparib (50 mg/kg) daily from day 1 to day 6, and irradiated (dose per fraction, 2 Gy) on days 1, 2, 4 and 5. Analyses of tumour growth (left panel), mouse survival (middle), body weight (right panel) and statistical analysis of mouse survival (bottom) are shown.

At a lower dose of irradiation (4*2 Gy), concomitant treatment with 50 mg/kg olaparib augmented the antitumour efficacy of RT against subcutaneous LL2-luc tumours (Fig. 3a), in agreement with previously reported studies evaluating the therapeutic efficacy of PARP inhibitors in combination with the standard 2-Gy dosefractionated irradiation regimen against human subcutaneous tumours.^14,16,18,25,28,31^ In contrast, the combination of 50 mg/kg olaparib with 4 fractions of 2 Gy was not more effective than IR alone against orthotopic LL2-luc tumours, though no weight loss was observed under these treatment conditions (Fig. 3b). Thus, the therapeutic window to treat lung cancers with the olaparib and IR combination treatment might be narrower than expected.

### The olaparib plus IR combination treatment triggers ATR/Chk1 signalling

**Fig. 4:**
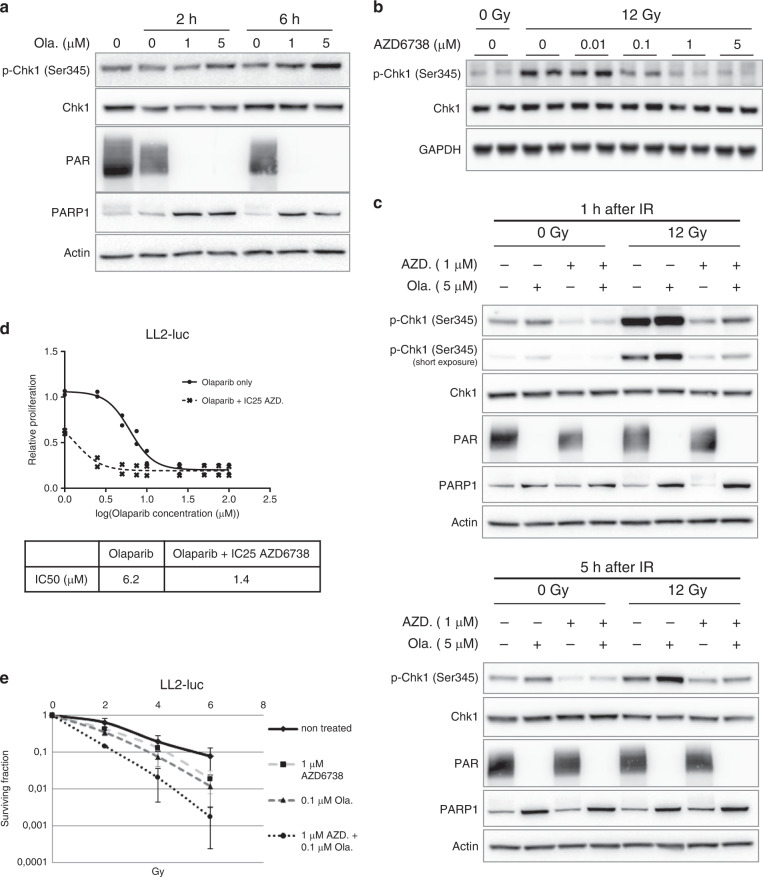
ATR inhibition eradicates the olaparib-induced activation of ATR/Chk1 signalling, and sensitises tumour cells to olaparib and/or IR. **a** Western blot analysis of Chk1 phosphorylation after 2 h or 6 h of olaparib (Ola.) treatment. **b** AZD6738 treatment inhibits radio-induced Chk1 phosphorylation. LL2-luc cells were treated with AZD6738 for 1 h before irradiation (12 Gy). Protein extracts were prepared at 30 min after irradiation. **c** Western blot analysis of LL2-luc cells treated with AZD6738 (AZD.) and/or olaparib (Ola.) for 1 h before irradiation (12 Gy). Protein extracts were prepared at 1 h or 5 h after irradiation. **d** The proliferation of LL2-luc cells treated or not with increasing doses of olaparib and with 0.6 µM AZD6738 (corresponding to the IC_25_) was analysed by WST-1 assay. **e** Clonogenic survival of LL2-luc cells exposed concomitantly to olaparib, AZD6738 and IR. Cells were treated with both compounds and irradiated 1 h later, and the drugs were removed 23 h later.

Unrepaired SSBs and PARP–DNA-trapping lesions triggered by olaparib treatment increased ATR/Chk1 signalling, as demonstrated by the increased phosphorylation of Chk1 after olaparib treatment (Fig. 4a), consistent with previous studies.^12,35,36^ Chk1 phosphorylation was also augmented by IR alone (Fig. 4b), and was more pronounced in cells treated with both olaparib and IR than in those treated with either single agent alone (Fig. 4c). Treatment with the AZD6738 ATR inhibitor completely inhibited Chk1 activation in irradiated cells (Fig. 4b). AZD6738 alone showed anti-proliferative effects and radiosensitising properties (Supplementary Fig. 3a, b) against LL2-luc cells. AZD6738 also eradicated olaparib-induced Chk1 phosphorylation, regardless of whether the cells were irradiated (Fig. 4c), suggesting a potential role for the ATR/Chk1 signalling pathway in the survival of olaparib-treated and olaparib + IR-treated cells. Consistently, AZD6738 sensitised LL2-luc tumour cells to the olaparib treatment (Fig. 4d) and the olaparib plus IR combination treatment (Fig. 4e), prompting the evaluation of the triple combination in vivo.

### The antitumour efficacy of the olaparib, AZD6738 and IR triple-combination treatment varies with tumour location

**Fig. 5:**
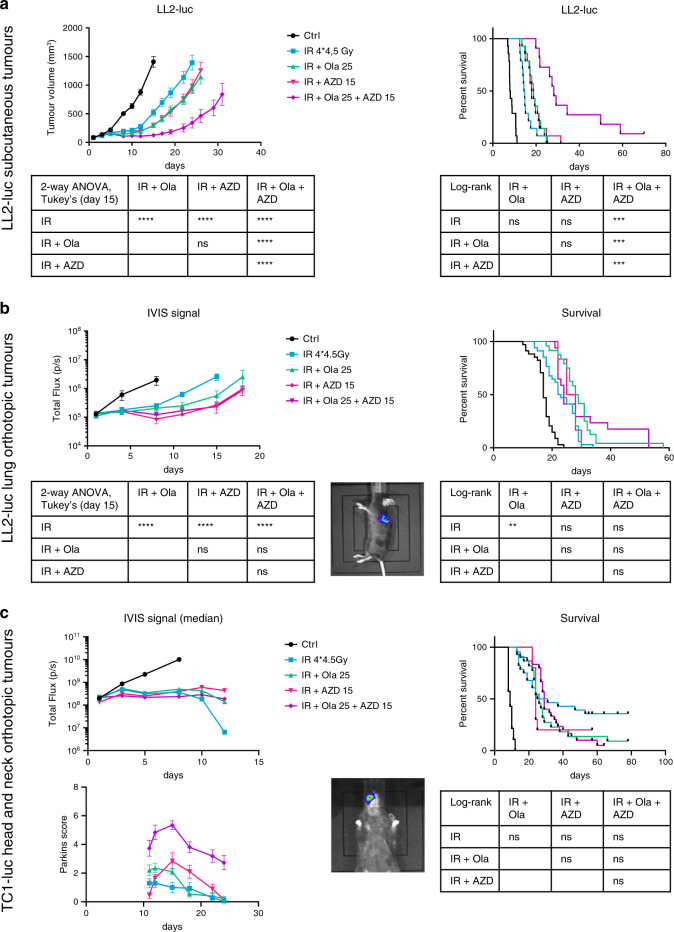
The AZD6738 ATR inhibitor enhances the antitumour efficacy of the olaparib plus IR combination treatment in subcutaneous grafts, but not in lung or head and neck orthotopic tumours. Tumour-bearing mice were allocated to treatment groups on day 1. Olaparib (25 mg/kg) and/or AZD6738 (15 mg/kg) were administered daily from day 1 to day 6. Irradiated mice received four fractions of 4.5 Gy (local irradiation on days 1, 2, 4 and 5). **a** LL2-luc subcutaneous grafts (*n* = 11–14). **b** LL2-luc lung orthotopic tumours (representative image of bioluminescence capture in the middle of the panel, *n* = 6–34). **c** TC1-luc head and neck orthotopic tumours (representative image of bioluminescence capture in the middle of the panel, *n* = 10–30). For **a**, **b** and **c**, tumour growth (upper- left panel, mean ± SEM or median), Kaplan–Meier analysis of mouse survival (upper-right panel) and statistical analyses (bottom) are depicted. For **c**, some mice were additionally scored for mucositis using a macroscopic scoring system (mean ± SEM, *n* = 10–13, lower-right panel).

As previously reported for subcutaneous tumours,^17,19,24,29^ ATR inhibition sensitised LL2-luc subcutaneous tumours to IR (Fig. 5a). The addition of AZD6738 to the olaparib plus IR treatment further delayed tumour growth and prolonged survival compared with the effects of double-agent treatments (Fig. 5a). However, the triple combination was not more effective than the double-agent treatment against orthotopic LL2-luc lung tumours (Fig. 5b). In the triple-combination treatment, mice were prostrated and weak and lost weight around day 10 (Supplementary Fig. 4). As chest irradiation can be considered a non-localised irradiation, we investigated another orthotopic model, a mouse luciferase-expressing TC1-luc head and neck tumour model.^33^ We assessed whether TC1-luc cells were sensitive to olaparib and AZD6738 treatments in vitro in the same concentration range as LL2-luc cells (Supplementary Fig. 5a, b). AZD6738 treatment did inhibit the olaparib-induced activation of Chk1 in irradiated and nonirradiated TC1-luc cells (Supplementary Fig. 5c). Finally, AZD6738 sensitised TC1-luc cells to olaparib and olaparib plus IR treatments in vitro (Supplementary Fig. 5d, e). As observed for LL2-luc subcutaneous tumours, the triple-combination treatment (IR + olaparib + AZD6738) was more effective than the double-agent treatments against TC1-luc subcutaneous tumours (Supplementary Fig. 6). The irradiation of TC1-luc head and neck tumours with four fractions of 4.5 Gy greatly improved mouse survival. Ten out of 28 mice were completely cured. Surprisingly, the addition of a concomitant treatment with olaparib, AZD6738 or both olaparib and AZD6738 did not improve the antitumour efficacy of RT, and tended to worsen mouse fate (with only two out of 22 mice, two out of 10 mice and two out of 30 mice being cured in the irradiation + olaparib, irradiation + AZD6738 and irradiation + olaparib + AZD6738 treatment groups, respectively) (Fig. 5c, upper panels). A decrease in body weight was not observed for treated mice, but some mice showed signs of mucositis, a well-known side effect of irradiation. This acute reaction was scored macroscopically using Parkins’ scoring system (Table 1).^37,38^ Mice treated with the triple combination, and to a lesser extent, with the double combinations, developed a severe lip mucosal/epidermal reaction (Fig. 5c, lower panel), highlighting the toxicity elicited by these treatments.

**Table 1:** Parkins’ scoring system for lip reaction in mice (adapted from Parkins et al.^38^).

*Oedema score*	
0.5	Doubtful if any swelling
1	Slight but definite swelling
2	Severe swelling
3	Severe swelling and visible deformity
*Erythema/exudation score*	
0.5	Doubtful if abnormally pink
1	Slight but definite reddening
2	Severe reddening
3	Focal desquamation
4	Exudate or crusting involving approximately half of the lip area
5	Exudate or crusting involving more than half of the lip area

The Parkins’ score is the sum of the separate scores for oedema and erythema/exudation.

## Discussion

Our study reports clear differences in the response to antitumour treatments between mouse tumour models, and indicates that preclinical mouse models have to be well chosen to evaluate new therapeutic compounds.

To date, preclinical studies revealing the therapeutic efficacy of the IR plus PARP inhibition combination have been conducted in subcutaneous tumours, whether BRCA-deficient or BRCA-proficient, and the vast majority of these studies used a 2-Gy fractionated irradiation regimen.^14–16,18,20–23,25,28,31^ The benefit of this combination was confirmed in an orthotopic tumour model, a pancreatic xenograft of the MiaPaca-2 cell line.^30^ However, these studies were conducted with human tumour models grafted in immunocompromised mice. As a major role of the immune system in cancer control and treatment has been clearly highlighted, we aimed to explore the use of the olaparib plus IR combination against a syngeneic tumour model grafted in immunocompetent mice. We first observed that the standard 50 mg/kg olaparib dose improved the efficacy of IR against subcutaneous syngeneic tumours, as demonstrated in human subcutaneous xenografts, whether the tumours were irradiated with a standard 4*2-Gy dose or a higher dose of irradiation (4*4.5 Gy) (Figs. 1e and 3a). Surprisingly, the growth of the orthotopic lung tumour model was neither affected by four fractions of 2 Gy (perhaps as a consequence of faster tumour cell growth in this setting) nor by the concomitant addition of 50 mg/kg olaparib (Fig. 3a). We increased the radiation dose to four fractions of 4.5 Gy to induce a detectable effect of radiotherapy alone, and as olaparib worsens existing radio-induced DNA damage, we expected to observe an enhanced antitumour effect in the corresponding combination group. Surprisingly, the combination treatment was not more efficient than treatment with irradiation alone (Fig. 2a). At high doses of olaparib and IR, the mice experienced prostration and weakness and lost weight (Fig. 2a, c). Tolerability issues for the combination of olaparib and thoracic irradiation have been highlighted in a recent study: enhancement of radio-induced (4*5 Gy) acute toxicity was induced by concomitant treatment with 50 mg/kg olaparib in the highly replicative cells of the oesophagus, with a thickening of the keratin layer, epithelial hyperplasia and hypertrophy and persistent DSBs.^39^ Thus, normal oesophageal tissue toxicity might have affected mouse survival and tumour control, and may be one of the reasons for the lack of an impact of the olaparib plus IR combination in our orthotopic model. Nevertheless, the antitumour efficacy of four fractions of 4.5 Gy was improved by a concomitant treatment with the lower 25 mg/kg olaparib dose. Altogether, these results show that in the context of thoracic irradiation, the therapeutic window of the olaparib plus IR treatment may be very narrow. Consistent with these findings, a Phase 1 trial in breast cancer reported grade 3 adverse events for breast cancers treated with RT and the PARP inhibitor Veliparib.^40^ Toxicity issues for the combination of PARPi and RT are also evidenced by the lip epidermal/mucosal reaction observed in our head and neck tumour model (Fig. 5c). Notably, the incidence of mucositis was further increased by the addition of AZD6738, suggesting that patients treated with PARP and/or ATR inhibitor concomitantly with their radiotherapy course of treatment could be at higher risk for severe mucositis. Irradiation techniques used in our in vivo experiments are not as precise as those used in the clinic, and large parts of normal tissues are irradiated (oesophagus and a part of the liver in the lung orthotopic model and the entire oral mucosa in the head and neck orthotopic model). However, taken together, the data reported here, and in the literature, indicate that all highly proliferative tissues are likely to be extremely sensitive to therapeutic combinations of PARP inhibitors, and perhaps more generally, DDR inhibitors with radiotherapy. Although experiments conducted with other tumour cell lines, primary or autochthonous tumour models (to even better recapitulate the interaction between the tumour and microenvironment), and data from clinical trials are required to validate this hypothesis, we do believe that the transfer of these therapeutic combinations into the clinic might be carefully performed with well-chosen doses, and with particular attention to the reaction of the surrounding healthy tissue.

By including the stromal and immune environment, syngeneic orthotopic tumour models are thought to more closely resemble the complex process of human tumour progression and healthy tissue reaction. Orthotopic models generally grow faster, are more vascularised, are less hypoxic and are able to metastasise.^41–43^ Differences in the responses to antitumour treatment between subcutaneous and orthotopic tumours generated with the same tumour cell line have seldom been reported. In a lung tumour model, cisplatin but not mitomycin C exerted a significant antitumour effect against orthotopic tumours, reflecting the clinical situation, while subcutaneous tumours were sensitive to mitomycin C and less sensitive to cisplatin.^44^ Sensitivity to doxorubicin has also been described to depend on tumour location, with high sensitivity in subcutaneous sites and low sensitivity in the liver and in the lung, whereas the highest sensitivity to 5-FU was observed in the lung for the same tumour cell  line.^45^ Immunotherapy was shown to exert differential effects against murine orthotopic and subcutaneous tumours.^46–48^ Here, we showed that tumour location affects the efficacy of DDR inhibitors combined with IR in a murine lung tumour model and in a murine head and neck tumour model. Our results suggest that the limited efficacy of the olaparib + IR or olaparib + AZD6738 + IR combination treatments against LL2-luc and TC1-luc orthotopic tumours might be accompanied by tolerability issues. Experiments with the subcutaneous model failed to reveal tolerability issues and the narrowness of the therapeutic window, while such information might be essential for the potential transfer of these therapeutic strategies into the clinic.

The differential responses to the antitumour treatment observed between orthotopically and subcutaneously implanted tumours might be due in part not only to decreased tolerability but also to differences in vascularisation and tumour perfusion, as subcutaneous tumours are known to be less oxygenated than orthotopic tumours.^41–43^ Indeed, PARP inhibitors generally increase tumour vessel perfusion, and olaparib radiosensitises hypoxic rather than well-oxygenated tumours in vivo.^16,20,28^ Furthermore, lung orthotopic and subcutaneous implantations may not provide the same immune microenvironment, as previously demonstrated for other tumour sites, such as the colon or the kidney.^47,48^ Through the induction of extensive DNA damage, DDR inhibitors can modulate tumour inflammation, immune priming and immunosuppression.^49–51^ Thus, the differences in the response to the antitumour treatments in our study might also derive in part from differences in the reaction of the immune system across different tumour settings.

Our study reinforces the critical importance of using clinically relevant preclinical tumour models instead of the subcutaneous implantation model that is too often used. We think that syngeneic orthotopic tumours grafted in immunocompetent mice are far more informative to appropriately select drugs and design clinical trials, and we recommend the extensive use of these models in preclinical studies.

## Supplementary information

Supplementary information

## Data Availability

The data that support the findings of this study are available from the corresponding authors upon reasonable request.
