# Supplementary material for: Differential therapeutic effects of PARP and ATR inhibition combined with radiotherapy in the treatment of subcutaneous versus orthotopic lung tumour models
Source: Br J Cancer. 2020 Jun 17;123(5):762–71. doi: 10.1038/s41416-020-0931-6 (PMC7463250; doi:10.1038/s41416-020-0931-6)
Supplement: Supplementary file 1 — Supplementary information [file 41416_2020_931_MOESM1_ESM.pptx]

## Slide 1
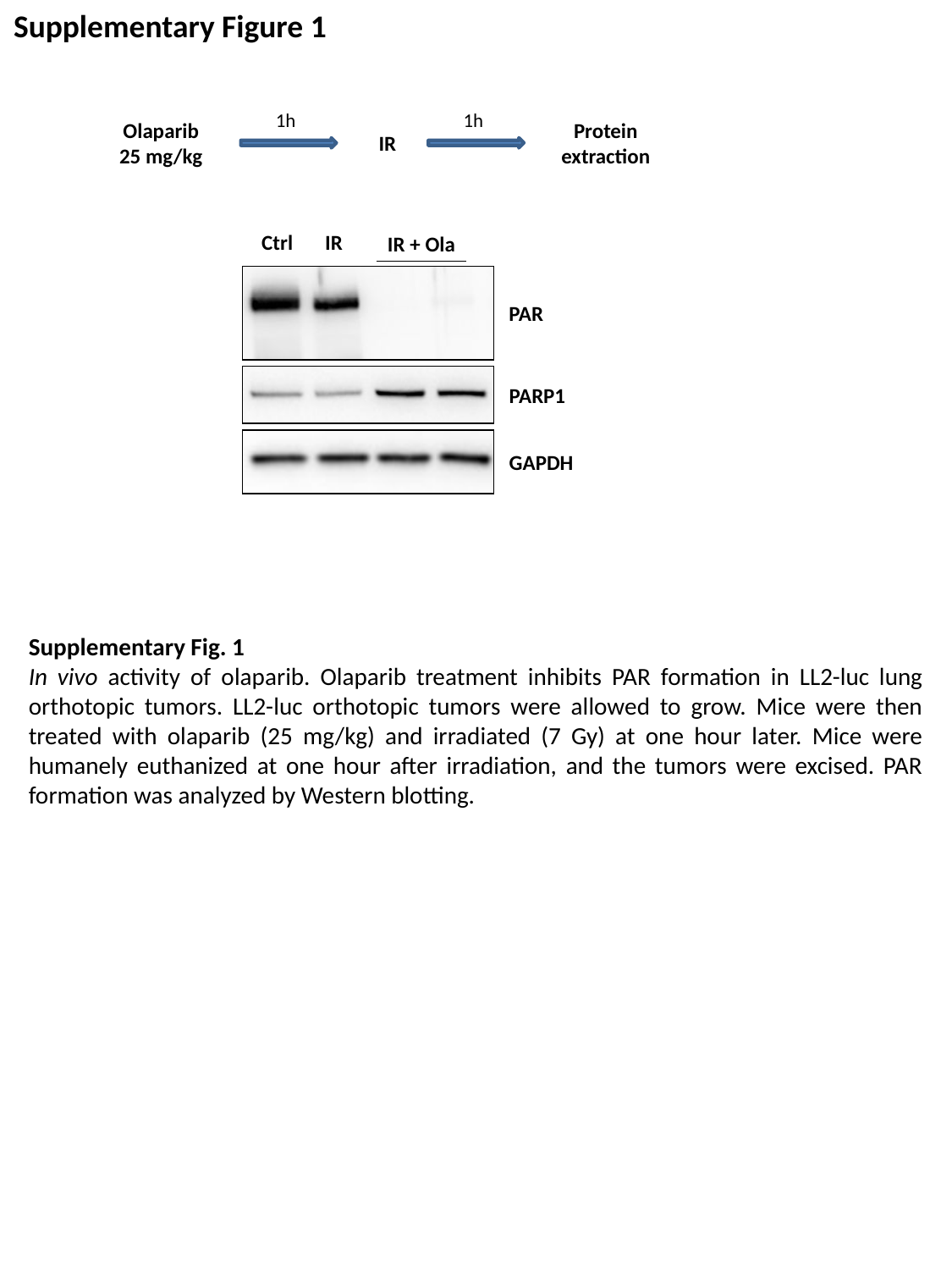

Supplementary Figure 1
1h
1h
Olaparib
25 mg/kg
Protein extraction
IR
Ctrl
IR
IR + Ola
PAR
PARP1
GAPDH
Supplementary Fig. 1
In vivo activity of olaparib. Olaparib treatment inhibits PAR formation in LL2-luc lung orthotopic tumors. LL2-luc orthotopic tumors were allowed to grow. Mice were then treated with olaparib (25 mg/kg) and irradiated (7 Gy) at one hour later. Mice were humanely euthanized at one hour after irradiation, and the tumors were excised. PAR formation was analyzed by Western blotting.

## Slide 2
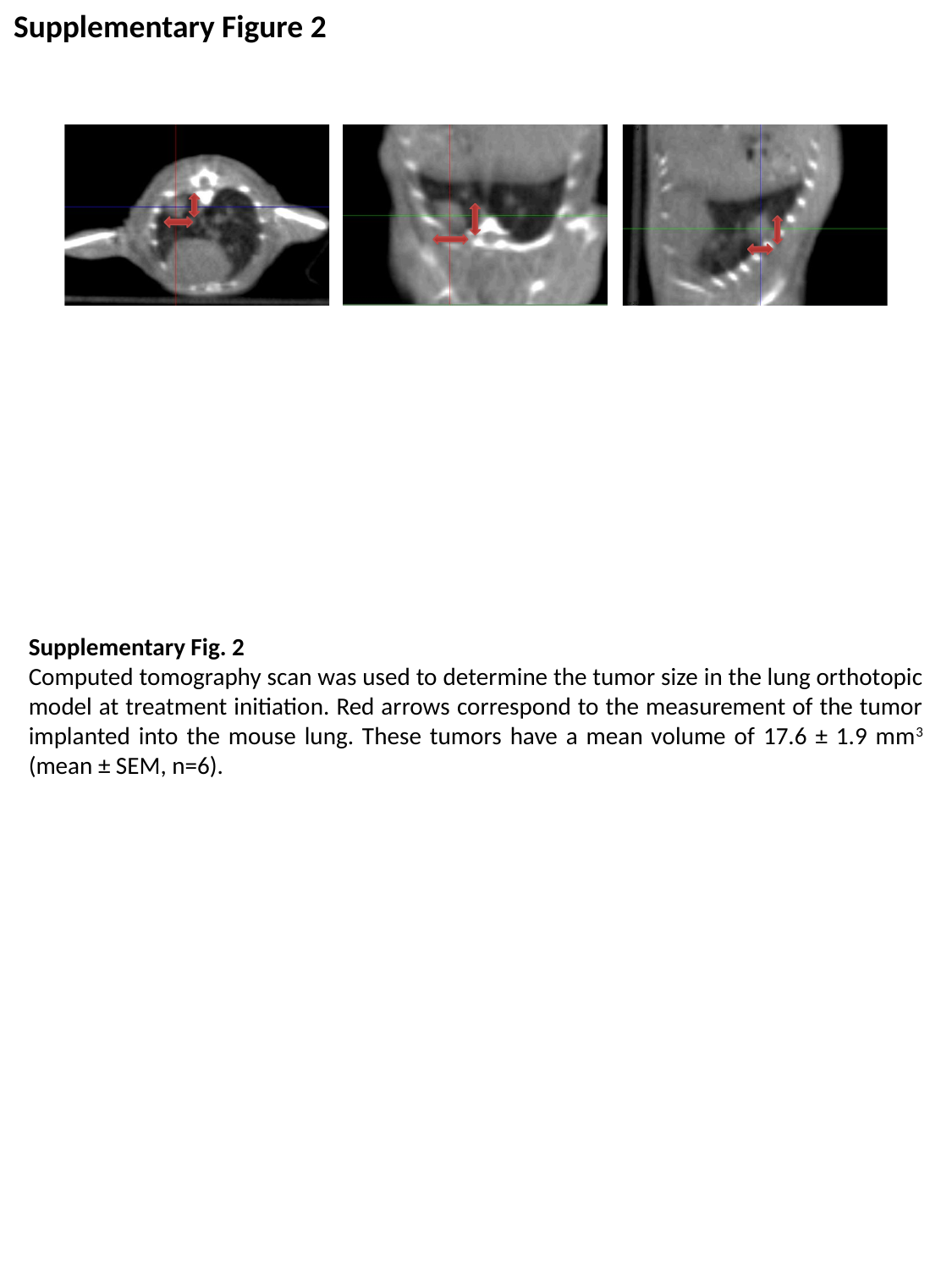

Supplementary Figure 2
Supplementary Fig. 2
Computed tomography scan was used to determine the tumor size in the lung orthotopic model at treatment initiation. Red arrows correspond to the measurement of the tumor implanted into the mouse lung. These tumors have a mean volume of 17.6 ± 1.9 mm3 (mean ± SEM, n=6).

## Slide 3
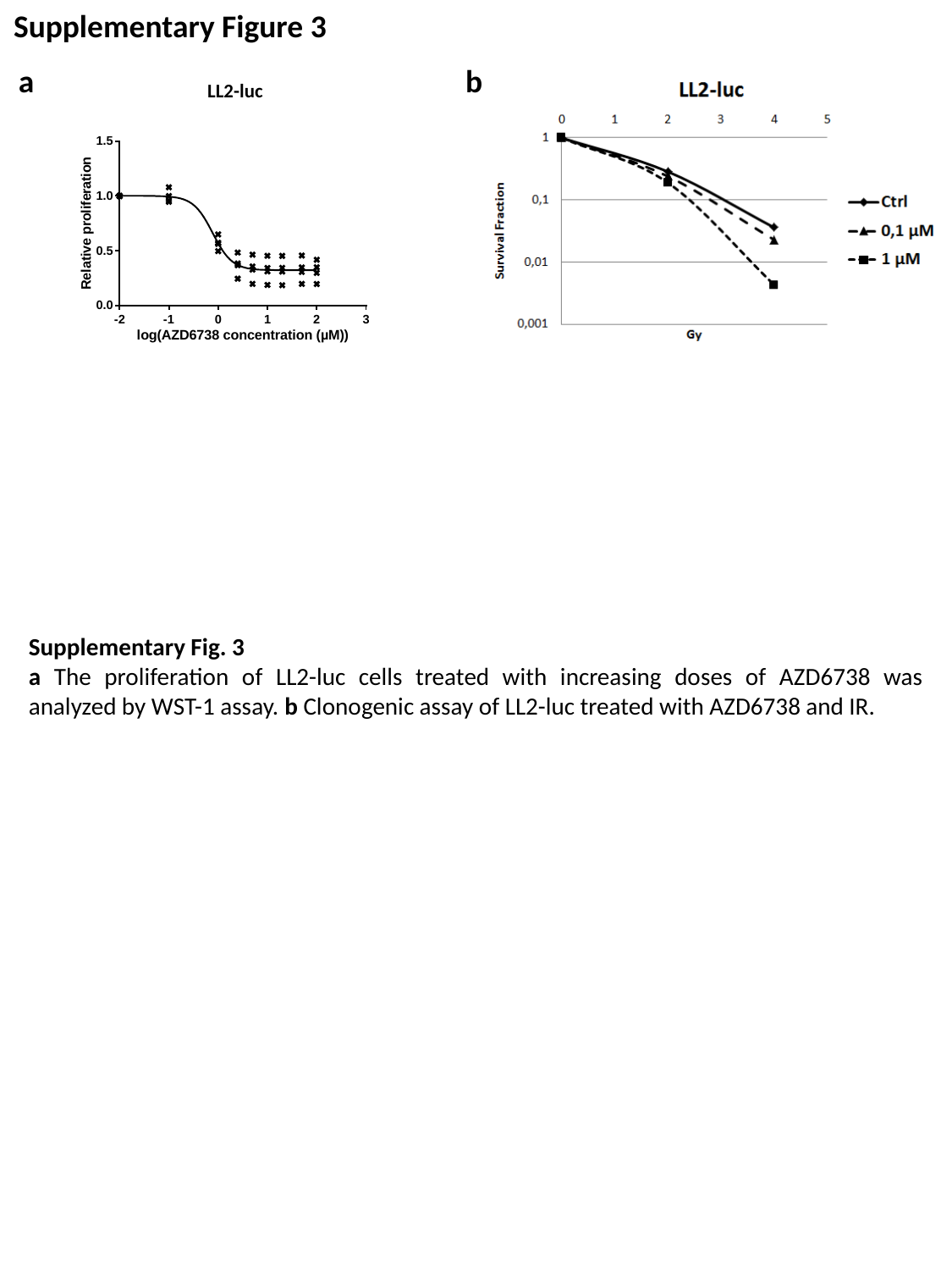

Supplementary Figure 3
a
b
LL2-luc
Supplementary Fig. 3
a The proliferation of LL2-luc cells treated with increasing doses of AZD6738 was analyzed by WST-1 assay. b Clonogenic assay of LL2-luc treated with AZD6738 and IR.

## Slide 4
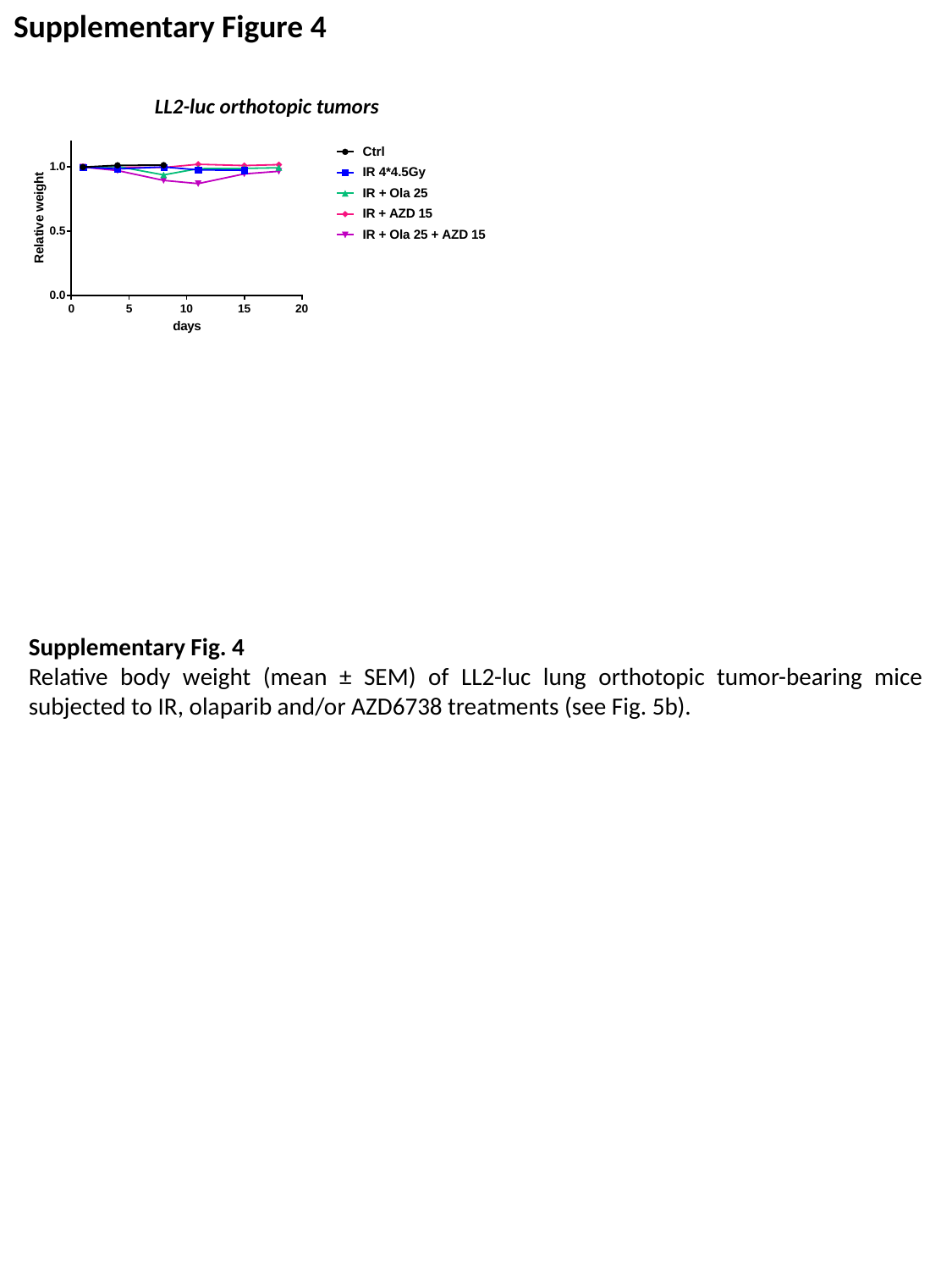

Supplementary Figure 4
LL2-luc orthotopic tumors
Supplementary Fig. 4
Relative body weight (mean ± SEM) of LL2-luc lung orthotopic tumor-bearing mice subjected to IR, olaparib and/or AZD6738 treatments (see Fig. 5b).

## Slide 5
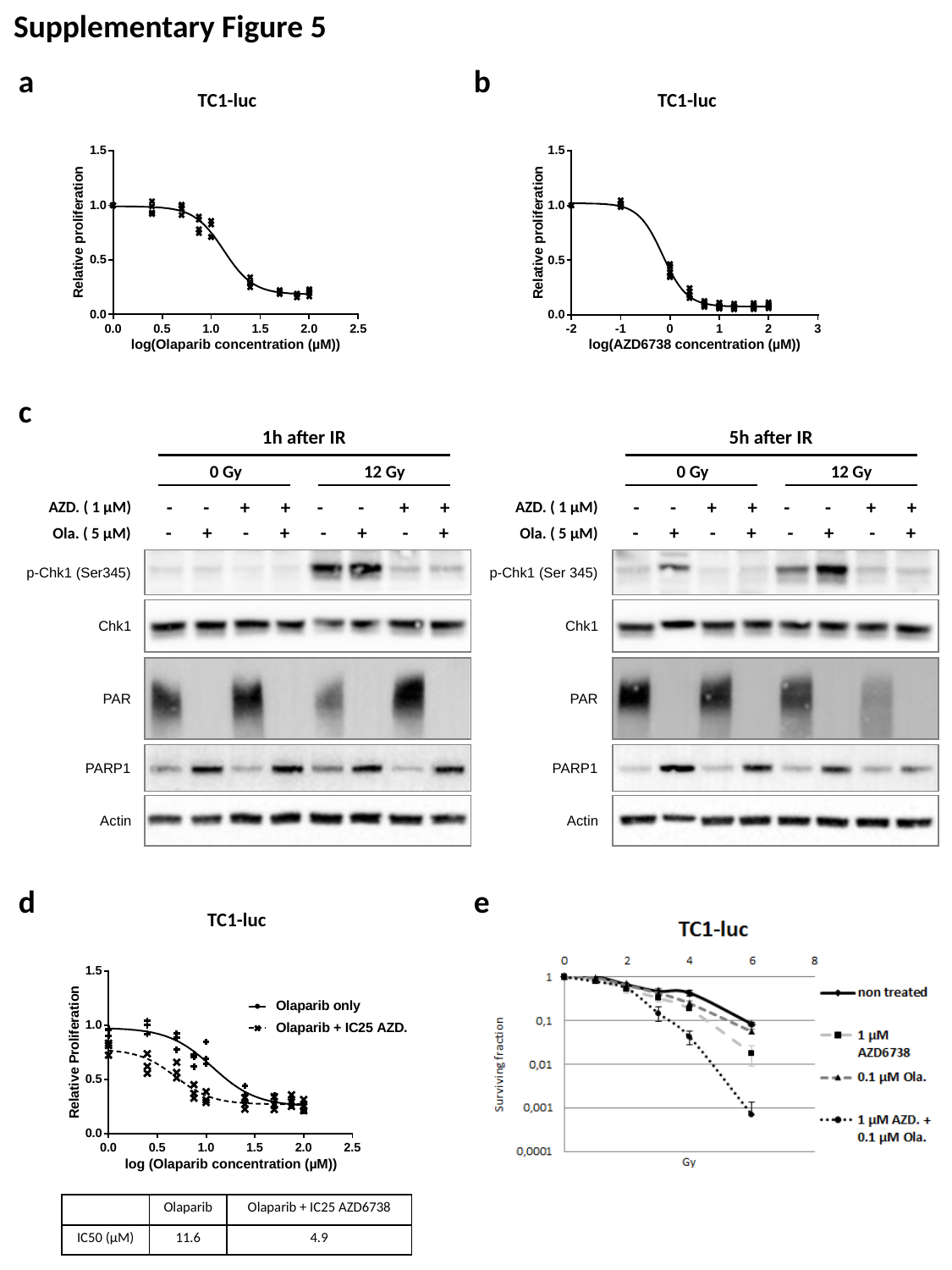

Supplementary Figure 5
a
b
TC1-luc
TC1-luc
c
1h after IR
0 Gy
12 Gy
- - + + - - + +
AZD. ( 1 µM)
- + - + - + - +
Ola. ( 5 µM)
p-Chk1 (Ser345)
Chk1
PAR
PARP1
Actin
5h after IR
0 Gy
12 Gy
- - + + - - + +
AZD. ( 1 µM)
- + - + - + - +
Ola. ( 5 µM)
p-Chk1 (Ser 345)
Chk1
PAR
PARP1
Actin
d
e
TC1-luc
| | Olaparib | Olaparib + IC25 AZD6738 |
| --- | --- | --- |
| IC50 (µM) | 11.6 | 4.9 |

## Slide 6
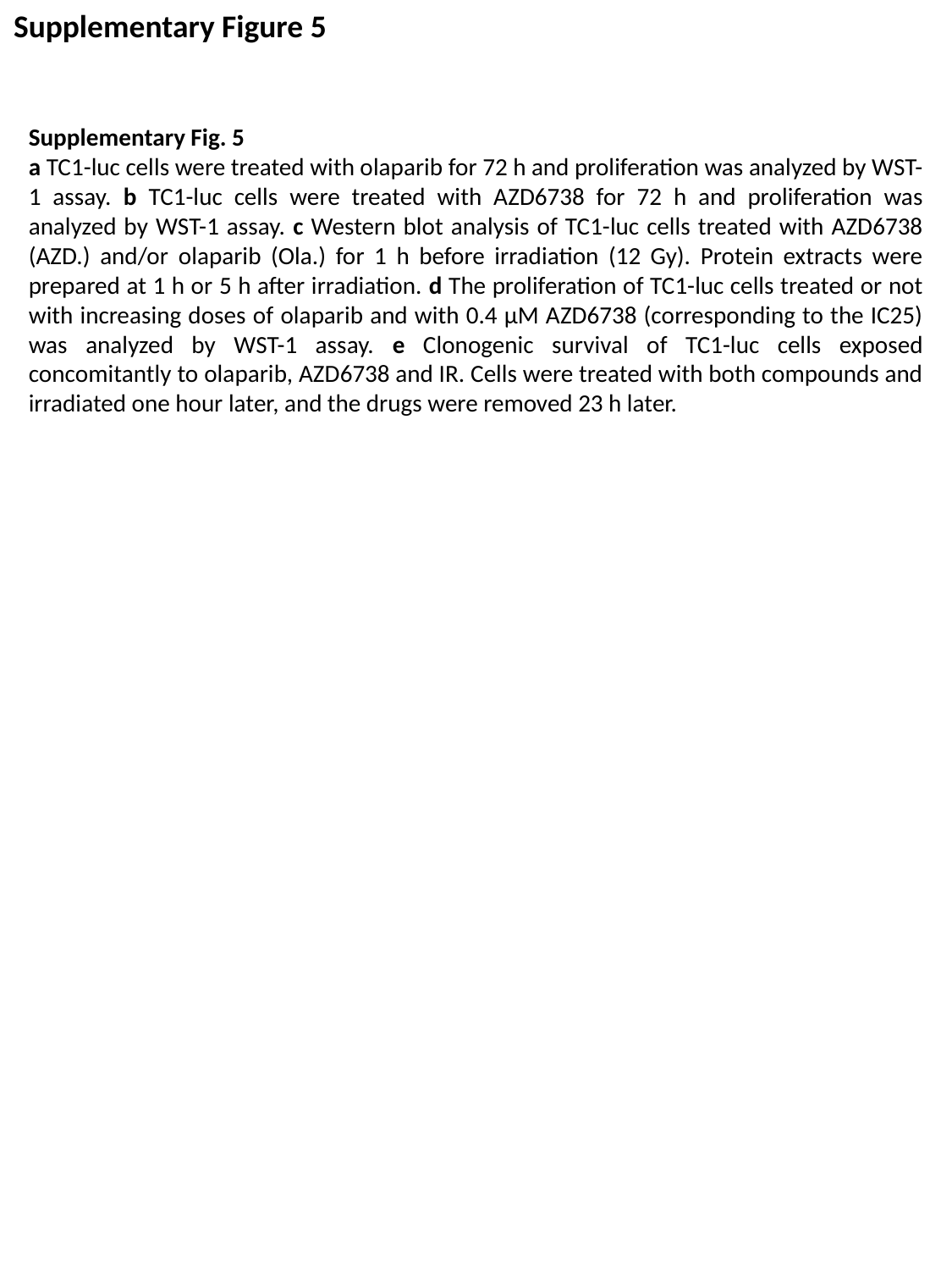

Supplementary Figure 5
Supplementary Fig. 5
a TC1-luc cells were treated with olaparib for 72 h and proliferation was analyzed by WST-1 assay. b TC1-luc cells were treated with AZD6738 for 72 h and proliferation was analyzed by WST-1 assay. c Western blot analysis of TC1-luc cells treated with AZD6738 (AZD.) and/or olaparib (Ola.) for 1 h before irradiation (12 Gy). Protein extracts were prepared at 1 h or 5 h after irradiation. d The proliferation of TC1-luc cells treated or not with increasing doses of olaparib and with 0.4 µM AZD6738 (corresponding to the IC25) was analyzed by WST-1 assay. e Clonogenic survival of TC1-luc cells exposed concomitantly to olaparib, AZD6738 and IR. Cells were treated with both compounds and irradiated one hour later, and the drugs were removed 23 h later.

## Slide 7
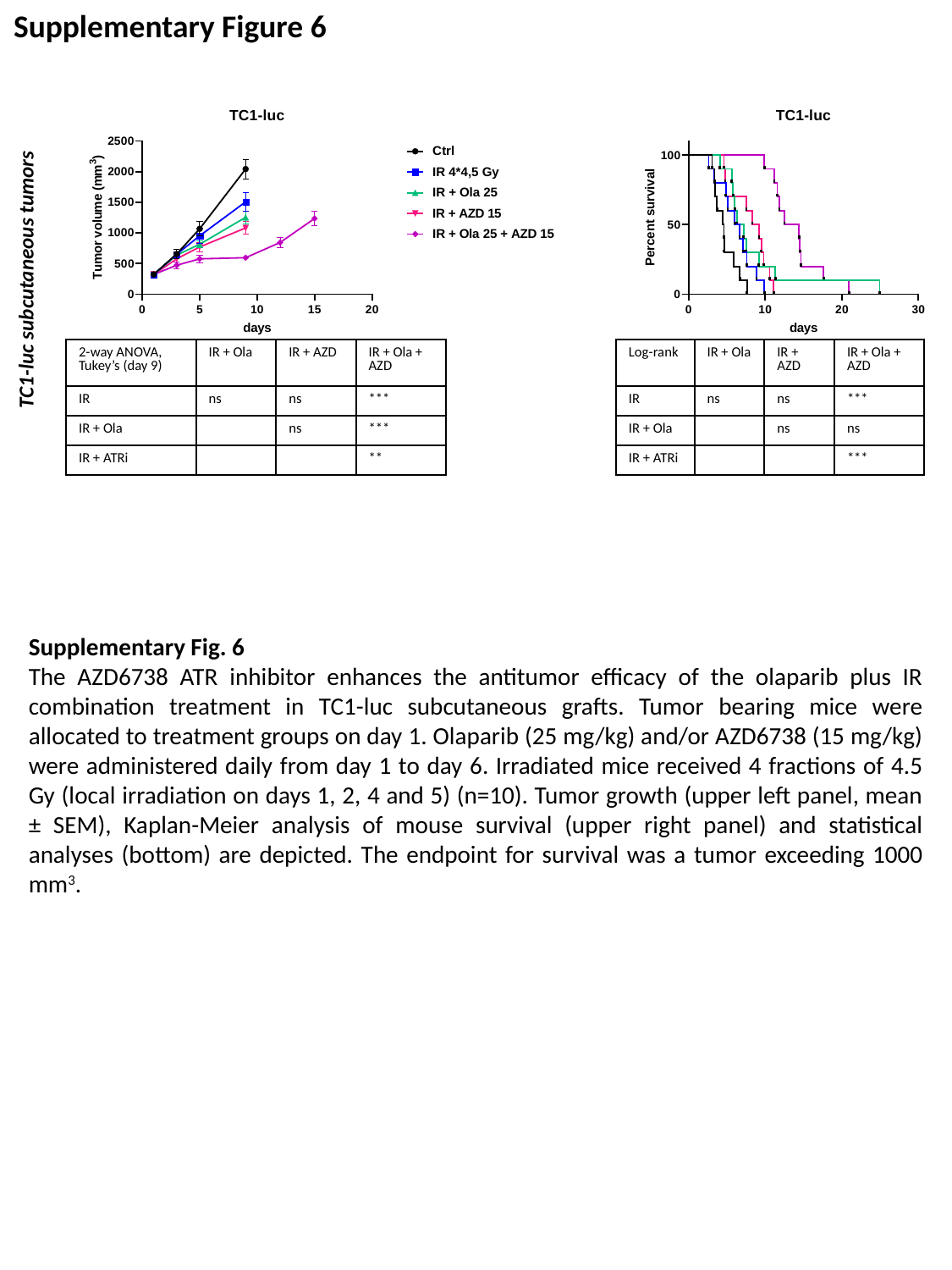

Supplementary Figure 6
TC1-luc subcutaneous tumors
| 2-way ANOVA, Tukey’s (day 9) | IR + Ola | IR + AZD | IR + Ola + AZD |
| --- | --- | --- | --- |
| IR | ns | ns | \*\*\* |
| IR + Ola | | ns | \*\*\* |
| IR + ATRi | | | \*\* |
| Log-rank | IR + Ola | IR + AZD | IR + Ola + AZD |
| --- | --- | --- | --- |
| IR | ns | ns | \*\*\* |
| IR + Ola | | ns | ns |
| IR + ATRi | | | \*\*\* |
Supplementary Fig. 6
The AZD6738 ATR inhibitor enhances the antitumor efficacy of the olaparib plus IR combination treatment in TC1-luc subcutaneous grafts. Tumor bearing mice were allocated to treatment groups on day 1. Olaparib (25 mg/kg) and/or AZD6738 (15 mg/kg) were administered daily from day 1 to day 6. Irradiated mice received 4 fractions of 4.5 Gy (local irradiation on days 1, 2, 4 and 5) (n=10). Tumor growth (upper left panel, mean ± SEM), Kaplan-Meier analysis of mouse survival (upper right panel) and statistical analyses (bottom) are depicted. The endpoint for survival was a tumor exceeding 1000 mm3.
